# Supplementary material for: Lessons from piloting and scaling a real-time DHIS2 based treatment reporting tool for mass drug administration in Nigeria
Source: PLoS Negl Trop Dis. 2025 Oct 10;19(10):e0013601. doi: 10.1371/journal.pntd.0013601 (PMC12530614; doi:10.1371/journal.pntd.0013601)
Supplement: S1 File — It also contains assessment results from different states regarding ownership measurements (Tables D, E & F), and participants’ responses presented in word clouds. These word clouds highlight the improvements and challenges related to data access, use, and quality based on a rapid survey conducted with research participants (Fig A). (DOCX) [file pntd.0013601.s001.docx]

## **Table A: Responsibility of each level of programme implementing teams before, during and after MDAs**

|  | **Before MDA** | **During MDA** | **After MDA** |
| --- | --- | --- | --- |
| LGA teams (M&E) | - Compile list of implementation units (IUs), including the health facilities - Compile list of community population and treatment targets for IUs - Share compiled lists with SMoH | - Data entry of community treatment and drug use/wastage into DHIS2 platform - Data fed into the DHIS2 dashboard (showing treatment numbers, progress against targets, and drug use to treatment ratio) | - Review meeting of implementation process - drug requisition, training of State and LGA teams, training of CDDs, DHIS2 training, drug distribution, data collection, entries into the DHIS2 platform, Command Centre monitoring during MDA, mop-up treatments for missed populations, and the effects of COVID-19 on treatment - Planning for the next MDA |
| State NTD team | - Review shared lists of community listing and treatment targets - Share lists with FMoH/Sightsavers | - Monitoring of DHIS2 dashboard and data quality checks/verification - Feedback to LGA teams via tool free helpline and WhatsApp chats |  |
| FMoH/Sightsavers | - Configure community forms into DHIS2 format - Clean lists and upload data into DHIS2 platform - Assign login details to M&E officers - Train LGA teams (two M&E officers per state) on use of DHIS2 platform - Set up of command center and train of state team |  |  |

## **Table B: Data Ownership Statements for assessing government ownership**

| **Components of government ownership** | **Statement** |
| --- | --- |
| **Data Access:**  *(Ability to view and get information that you need, such that you know what is happening during the MDA).* | During the MDA, I can see and track treatment coverage data at the community, LGA or State levels |
|  | During the MDA, there is a place/person I can go to access information about the MDA activities going on in my LGA/State. |
|  | Overall, I am happy with the level of access to information that I have during MDA in my LGA/State |
| **Data Use**  *(Ability to make evidence-based changes to the MDA program in order to improve the MDA activities whilst it is ongoing).* | During the MDA, I can use the information from the data to identify areas where improvements or changes may be needed. |
|  | During the MDA, I am able to make decisions and recommendations based on the data from the MDA. |
|  | Overall, I am satisfied with my ability to use the MDA data for identifying and making required programme changes before, during and after the MDA. |
| **Data control**  *(Is the ability to understand and take charge of the aspects of data entry, viewing or response that you are involved with during and after MDA)* | During the MDA, I understand the way the system of data entry/information and so can trouble shoot problems myself. |
|  | During the MDA, I feel I am in charge of the data from the communities, LGA or State. |
|  | Overall, I am satisfied with the level of control I have over the data from the MDA before, during and after the MDA exercise. |

## **Table C: Short open-ended questions for online pool**

| 1. | Where were the points that you came into contact with DHIS2 system during MDA this year? |
| --- | --- |
| 2a. | What has been some of the improvements to accessing MDA data via the DHIS2? |
| 2b. | What has been some of the downsides/challenges to accessing MDA data via the DHIS2? |
| 3b. | What has been some of the improvements to using MDA data obtained from the DHIS2? |
| 3c. | What has been some of the downside (drawbacks or difficulties) to using MDA data obtained via the DHIS2? |
| 4a. | What has been some of the improvements in the quality of MDA data from the DHIS2? |
| 4b. | What has been some of the downside (drawbacks or difficulties) in the quality of MDA data obtained via the DHIS2? |
| 5 | How do you think that the DHIS2 has impacted MDA overall (in terms of the coverage or speed of monitoring treatment coverage)? |

## **Table D: Ownership measurement in Kwara state**

| KWARA (49 responses) | Pre-intervention | Post-intervention | Pre vs Post score increase (t-test) |  |
| --- | --- | --- | --- | --- |
| MAIN AIM | Mean scores for last MDA (max = 15points per component) | | | |
| Data Access | 72% (10.8/15) | 84% (12.6/15) | t=3.16, p=0.002 |  |
| Data Use | 77% (11.6/15) | 85% (12.7/15) | t=1.84, p=0.068 |  |
| Data Control | 71% (10.7/15) | 86% (12.9/15) | t=3.77, p=0.003 |  |
| SUB-AIM | Expectation  (Pre-Int: next MDA) | Reality  (Post-Int: last MDA) | Difference in scores  (Expectation- Reality) |  |
| Data Access | 94% (14.1/15) | 84% (12.6/15) | 10% |  |
| Data Use | 93% (13.9/15) | 85% (12.7/15) | 8% |  |
| Data Control | 87% (13.1/15) | 86% (12.9/15) | 1% |  |

## **Table E: Ownership measurement in Enugu state**

| ENUGU (26 responses) | Pre-intervention | Post-intervention | Pre vs Post score increase (t-test) |
| --- | --- | --- | --- |
| MAIN AIM | Mean scores for last MDA (max = 15points per component) | | |
| Data Access | 69% (10.3/15) | 65% (9.7/15) | t=-0.63, p=0.534 |
| Data Use | 64% (9.6/15) | 62% (9.3/15) | t=-0.29, p=0.772 |
| Data Control | 61% (9.1/15) | 57% (8.5/15) | t=-0.49, p=0.630 |
| SUB-AIM | Expectation  (Pre-Int: next MDA) | Reality  (Post-Int: last MDA) | Difference in scores  (Expectation- Reality) |
| Data Access | 97% (14.5/15) | 65% (9.7/15) | 32% |
| Data Use | 97% (14.8/15) | 62% (9.3/15) | 35% |
| Data Control | 95% (14.2/15) | 57% (8.5/15) | 38% |

## **Table F: Ownership measurement in Jigawa state**

| JIGAWA (54 responses) | Pre-intervention | Post-intervention | Pre vs Post score increase (t-test) |
| --- | --- | --- | --- |
|  | Mean scores for last MDA (max = 15points per component) | | |
| Data Access | 91% (13.6/15) | 89% (13.3/15) | t=-0.77, p=0.440 |
| Data Use | 86% (12.8/15) | 89% (13.3/15) | t=0.88, p=0.378 |
| Data Control | 83% (12.4/15) | 92% (13.8/15) | t=2.60, p=0.011 |
| SUB-AIM | Expectation  (Pre-Int: next MDA) | Reality  (Post-Int: last MDA) | Difference in scores  (Expectation- Reality) |
| Data Access | 91% (13.6/15) | 89% (13.3/15) | 2% |
| Data Use | 86% (12.9/15) | 89% (13.3/15) | -3% |
| Data Control | 81% (12.1/15) | 92% (13.8/15) | -11% |

***
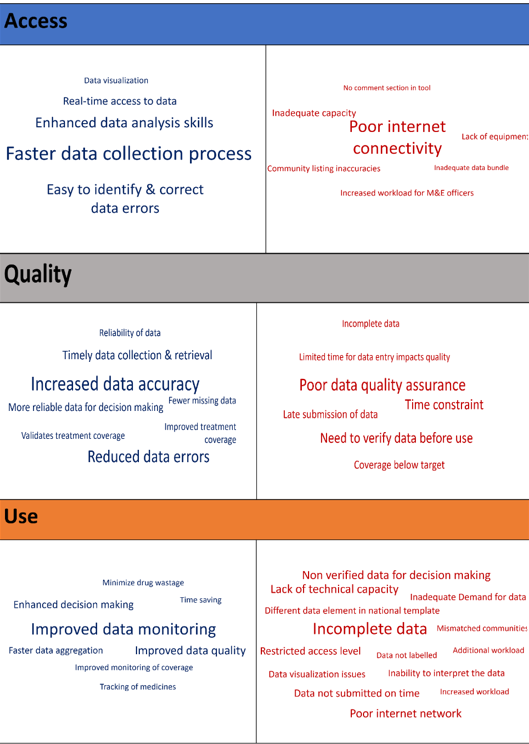
***

## ***Fig A: Word-cloud for improvement and challenges with data access, use and quality in the rapid survey***
