# Supplementary material for: Lessons from piloting and scaling a real-time DHIS2 based treatment reporting tool for mass drug administration in Nigeria
Source: PLoS Negl Trop Dis. 2025 Oct 10;19(10):e0013601. doi: 10.1371/journal.pntd.0013601 (PMC12530614; doi:10.1371/journal.pntd.0013601)
Supplement: Attachment — Submitted filename: Major comments_25-00416.docx [file pntd.0013601.s002.docx]

Comments to authors (PNTD-D-25-00416):

Using paper-based systems for mananging mass drug administration (MDA) campaings for neglected tropical diseases (NTDs) can lead to several operational and strategic challenges, thereby hindering process toward NTD elimination targets. This manuscript reports a timely study that offers insights into the digital transformation of MDA implementation.

This manuscript reports on the use of the DHIS2 system for MDA implementation in the settings with high disease burden of neglected tropical diseases. The study used a range of qualitative methods, including field dairies, on users exepirences. Uses from implementation units, states and command center reported positive feedback on data accessibility, data quality and data ownership. Such improvements include increased, faster and unrestricted access, affectives responses from users, timely identification of data error types, and government ownership expectation, etc.

The evaluation of DHIS2 relied heavily on qualitative analysis, which may be adequate if the primary focus is on user experience, system usability or acceptability. However, the authored mentioned treatment and stocking reporting in the title, and stated aspects such as timeliness, quality and treatment data availability, and centralized storage and control in the introduction section. Drug outstock, usage and wastages were aslo mentioned in the introduction, yet most ot these outcomes were not assessed by objective data. Instead, they were evaluated through user-reported perceptions and subjective indicators.

Drug stockouts, overstocking or wastage, delayed or missed treatment cycles, inaccurate coverage reporting, and operational costs are well-known challenge in MDA implementation. Relying on users’ perception of the system improvement is insufficient to assess the DHIS2 platform effectively. Furthermore, DHIS2 was known as a health management information system. The authors should also clarity how the system was used in logistics management or stock controlling in this context.

In terms of writing and presentation, there are occasional inconsistencies between what is expected and what is analysed. Aligning these more closely would strengthen the manuscript coherence.

General comments:

- The title of this manuscript is somewhat ambituous and overly general, lacking clarity on specific outccome assessed or the study design (a pre-post comparison). It is strongly suggested that the authors reivse the title accordingly and consider following relevant reporting guidelines such as GRAMMS or COREQ.
- The title claims DHIS2 was used for both treatment and stock reporting, but the data on stock (inventory, resupply, redistribution) is limited. While the manuscript describes the use of DHIS2 for tracking treatment and stock data, it is essential to clarify that DHIS2 is primarily a Health Management Information System (HMIS) not a dedicated logistics management information system (LMIS).
- While the abstract summarizes several positive outcomes, such as increased data use and improved government ownership, and enhanced decision-making, the description remains quite general. There is no quantitative estimate of these improvements, which limits the reader’s ability to assess the magnitude or significance. Including brief quantitative indicators would strengthen the abstract.
- The result section provides a structured narrative summary of the focus group discussion, with defined themes and participants quotations. A high-quality result section should also include interpretive narrative that synthesizes the data and explains how the quotes support each theme. The author should consider the following suggestions to strengthen the presentation: a) clarity theme development, b) indicate number of coders and coding process, c) codes link to the quotations, etc.
- In the discussion highlights the improvements in data quality due to real-time feedback. However, the DHIS2 systems’s potential to include automated validation checks on range limits and logical consistency is underexplored. How theses features could be leveraged or enhanced in future scale-up efforts should be discussed.
- In terms of formatting and design, tables containing vertical lines are generally avoided in academic publications to improve readability. The authors should consider using only horizontal lines and spacing to delineate rows and headers, in line with standard formatting guidelines. There is redundancy in the ‘state’, ‘region’, ‘type of MDA’ column. Please consider grouping rows for the same state and using a multi-row cell for shared values.

Major comments:

- The author mentioned key indicators such as treatment coverage, drug usage and wastage. However, it is unclear why these indicators were not campared before and after the introduction of the DHIS2 system, especailly given that DHIS2 system was used as a real-time treatment and stock reporting tool.
- In table 2, the sample size appears to reflect multiple typees of unit, such as data points, participants and activity accounts. However, the unit were not stated clearly. I feel it is inappropriate to merge information in such a table.
- line 374: Table 4 presents important comparative data, but the current table format is text-heavy and difficult to interpret quicly. It is recommended that reformatting the table to enhance its effectiveness and readability. For example, consider adding column headers to distinguish different time periods, and grouping rows with geographic area to improve visual organization.

Minor comments:

- p.1, line 28: The term ‘study sites’ is used to refer to entire states in the manuscript. Please replace it with ‘study area’ or ‘study settings’ to improve the geographic specificity of the study. Study sites are typically used to describe localized location such as hospitals, clinics or communities.
- p.1, line 37-41: the mention of ‘similar benefits across all models of partner assistance’ would be more informative if the models were briefly characterized.
- p.18, line 675: In table 9, the background color of the first column/row significantly reduces legibility. It is recommended that the authors use higher contrast colors or removing the background shadings altogether to ensure the table remains clearly legible.
- p.5, line 133: (page nubmer is inconsistent-after page 4, this following page is labeled as page1) In table 1, it is suggested that the authors using only horizontal lines and spacing to delineate rows and headers, in line with standard formatting guidelines. Also, there is redundancy in the ‘state’, ‘region’, ‘type of MDA’ column. Please consider grouping rows for the same state and using a multi-row cell for shared values. Alternatively, the authors could move the “Type of MDA” column to the first column, which could distinguish between different diseases being targeted across the same states.
- line 135: To enhance understanding of how the DHIS2 platform was used across adminstrative levels, the authors could consider including a diagram illustrating the health information system architecture--linking system core components, user roles, data flow or database involved.
- line 140: Could the authors please explain why local government area (LGA) was presented under states. It should be helpful to briefly explain this administrative structure for international reader to improve contextual understanding.
- line 347: in figure 1, the figure is titled to report number/frequency, but it displays percentages. It is recommended that the authors clarify what activity was measured as ‘access’ (log-in, form submission, or report generation?). If possible, use a stacked bar chart to show the total platform engagement broken down by activity or user type.
