# Supplementary material for: Lessons from piloting and scaling a real-time DHIS2 based treatment reporting tool for mass drug administration in Nigeria
Source: PLoS Negl Trop Dis. 2025 Oct 10;19(10):e0013601. doi: 10.1371/journal.pntd.0013601 (PMC12530614; doi:10.1371/journal.pntd.0013601)
Supplement: Attachment — Submitted filename: Review comments.docx [file pntd.0013601.s003.docx]

Dear Editors and Reviewers:

Review of Evaluating DHIS2 as a real-time treatment and stock reporting tool for mass drug administration in Nigeria due to PLOS Neglected Tropical Diseases：The manuscript number is PNTD-D-25-00416,the overall content of the article was good, and the DHIS2 system was able to address the problem of communities using a variety of paper matrices to organize data from the community, which are then transferred to the local government level and then to the district, and finally aggregated to the national level. It was also able to enable many NTD programs to access the timeliness, quality, and availability of comprehensive treatment data during and after MDA campaigns, and can be effective in improving prevention and treatment coverage of neglected tropical diseases.

However, the article also has a bit of a flaw:

1. there are some problems with the formatting, such as the paragraphing on lines 570-572.

2. Another thing is that the references should be in “[]” format instead of “()”, and the format of references should be standardized.

3. At the same time in the article inside the table should be simple and clear, will be some important form of content for the condensation of the table, at the same time will be the table inside the key information for the notes and explanations (such as “*Refresher training” is not explained).

4. references should be adequate so that they strongly support your article.

5. In the study, only 3 states (Jigawa, Enugu, Kwara) are covered, can you state whether this is representative of the diversity of Nigeria as a whole (e.g. difference between North and South). In terms of questionnaire completion: the sample size of the self-administered questionnaire was uneven across states (Kwara=49, Enugu=26), which may affect the credibility of the inter-state comparisons.

Sincerely,

Smile&jy
