# Supplementary material for: Lessons from piloting and scaling a real-time DHIS2 based treatment reporting tool for mass drug administration in Nigeria
Source: PLoS Negl Trop Dis. 2025 Oct 10;19(10):e0013601. doi: 10.1371/journal.pntd.0013601 (PMC12530614; doi:10.1371/journal.pntd.0013601)
Supplement: Attachment — Submitted filename: Responses to reviewers.docx [file pntd.0013601.s005.docx]

Thank you to the reviewers for their extremely useful feedback. Please find our responses in blue below each point.

**Comments to authors (PNTD-D-25-00416) (from Word document)**

Using paper-based systems for managing mass drug administration (MDA) campaigns for neglected tropical diseases (NTDs) can lead to several operational and strategic challenges, thereby hindering process toward NTD elimination targets. This manuscript reports a timely study that offers insights into the digital transformation of MDA implementation.

This manuscript reports on the use of the DHIS2 system for MDA implementation in settings with high disease burden of neglected tropical diseases. The study used a range of qualitative methods, including field dairies, on users’ experiences. Uses from implementation units, states and command center reported positive feedback on data accessibility, data quality and data ownership. Such improvements include increased, faster and unrestricted access, effectives responses from users, timely identification of data error types, and government ownership expectation, etc.

The evaluation of DHIS2 relied heavily on qualitative analysis, which may be adequate if the primary focus is on user experience, system usability or acceptability. However, the authored mentioned treatment and stocking reporting in the title, and stated aspects such as timeliness, quality and treatment data availability, and centralized storage and control in the introduction section. Drug out stock, usage and wastages were also mentioned in the introduction, yet most of these outcomes were not assessed by objective data. Instead, they were evaluated through user-reported perceptions and subjective indicators.

Drug stockouts, overstocking or wastage, delayed or missed treatment cycles, inaccurate coverage reporting, and operational costs are well-known challenges in MDA implementation. Relying on users’ perception of the system improvement is insufficient to assess the DHIS2 platform effectively. Furthermore, DHIS2 was known as a health management information system. The authors should also clarity how the system was used in logistics management or stock controlling in this context.

In terms of writing and presentation, there are occasional inconsistencies between what is expected and what is analysed. Aligning these more closely would strengthen the manuscript coherence.

General comments:

-The title of this manuscript is somewhat ambitious and overly general, lacking clarity on specific outcome assessed or the study design (a pre-post comparison). It is strongly suggested that the authors revise the title accordingly and consider following relevant reporting guidelines such as GRAMMS or COREQ.

--The title of the manuscript has been updated to align with study objectives and methodology.

-The title claims DHIS2 was used for both treatment and stock reporting, but the data on stock (inventory, resupply, redistribution) is limited. While the manuscript describes the use of DHIS2 for tracking treatment and stock data, it is essential to clarify that DHIS2 is primarily a Health Management Information System (HMIS) not a dedicated logistics management information system (LMIS).

--The title of the manuscript has been updated

-While the abstract summarizes several positive outcomes, such as increased data use and improved government ownership, and enhanced decision-making, the description remains quite general. There is no quantitative estimate of these improvements, which limits the reader’s ability to assess the magnitude or significance. Including brief quantitative indicators would strengthen the abstract.

--This point is well taken but the methods used in the study do not cater to brief quantitative indicators that can be shared. Instead, we have refocused the manuscript to focus on the lessons learned from the qualitative evaluation done.

-The result section provides a structured narrative summary of the focus group discussion, with defined themes and participants quotations. A high-quality result section should also include interpretive narrative that synthesizes the data and explains how the quotes support each theme. The author should consider the following suggestions to strengthen the presentation: a) clarity theme development, b) indicate number of coders and coding process, c) codes link to the quotations, etc.

--We have shortened the entire manuscript and shifted focus, so do not feel more information on the coding, etc would fit now.

-In the discussion highlights the improvements in data quality due to real-time feedback. However, the DHIS2 system’s potential to include automated validation checks on range limits and logical consistency is underexplored. How these features could be leveraged or enhanced in future scale-up efforts should be discussed.

--This is included as a recommendation in the Discussion under “Enhancement to the tool”.

-In terms of formatting and design, tables containing vertical lines are generally avoided in academic publications to improve readability. The authors should consider using only horizontal lines and spacing to delineate rows and headers, in line with standard formatting guidelines. There is redundancy in the ‘state’, ‘region’, ‘type of MDA’ column. Please consider grouping rows for the same state and using a multi-row cell for shared values.

--Thank you, the tables have been updated.

Major comments:

-The author mentioned key indicators such as treatment coverage, drug usage and wastage. However, it is unclear why these indicators were not compared before and after the introduction of the DHIS2 system, especailly given that DHIS2 system was used as a real-time treatment and stock reporting tool.

-- Our study was not designed as a comparative analysis. However, we included a comparison of coverage estimates solely to assess whether the figures obtained from DHIS2 aligned with programmatic CES benchmarks, as a proxy for data quality. Additionally, the component related to drug stock-taking has been removed, as it was not explored in this study.

-In table 2, the sample size appears to reflect multiple types of unit, such as data points, participants and activity accounts. However, the unit were not stated clearly. I feel it is inappropriate to merge information in such a table.

--Thank you, the tables have been updated.

-line 374: Table 4 presents important comparative data, but the current table format is text-heavy and difficult to interpret quickly. It is recommended that reformatting the table to enhance its effectiveness and readability. For example, consider adding column headers to distinguish different time periods, and grouping rows with geographic area to improve visual organization.

--Thank you, the tables have been updated.

Minor comments:

-p.1, line 28: The term ‘study sites’ is used to refer to entire states in the manuscript. Please replace it with ‘study area’ or ‘study settings’ to improve the geographic specificity of the study. Study sites are typically used to describe localized location such as hospitals, clinics or communities.

--All reference to ‘study sites’ have been removed

-p.1, line 37-41: the mention of ‘similar benefits across all models of partner assistance’ would be more informative if the models were briefly characterized.

--This has been revised and updated. We also provide a brief description of the various partnership models in the background.

-p.18, line 675: In table 9, the background color of the first column/row significantly reduces legibility. It is recommended that the authors use higher contrast colors or remove the background shadings altogether to ensure the table remains clearly legible.

--Thank you, the tables have been updated.

-p.5, line 133: (page number is inconsistent-after page 4, this following page is labeled as page1) In table 1, it is suggested that the authors using only horizontal lines and spacing to delineate rows and headers, in line with standard formatting guidelines. Also, there is redundancy in the ‘state’, ‘region’, ‘type of MDA’ column. Please consider grouping rows for the same state and using a multi-row cell for shared values. Alternatively, the authors could move the “Type of MDA” column to the first column, which could distinguish between different diseases being targeted across the same states.

--Thank you, the tables have been updated.

-line 135: To enhance understanding of how the DHIS2 platform was used across administrative levels, the authors could consider including a diagram illustrating the health information system architecture--linking system core components, user roles, data flow or database involved.

--This has been updated in the methods section.

-line 140: Could the authors please explain why local government area (LGA) was presented under states. It should be helpful to briefly explain this administrative structure for international readers to improve contextual understanding.

--

-line 347: in figure 1, the figure is titled to report number/frequency, but it displays percentages. It is recommended that the authors clarify what activity was measured as ‘access’ (log-in, form submission, or report generation?). If possible, use a stacked bar chart to show the total platform engagement broken down by activity or user type.

--Thank you, the figure has been updated

_____________________

**Reviewer feedback (from email)**

Dear Editors and Reviewers:

Review of Evaluating DHIS2 as a real-time treatment and stock reporting tool for mass drug administration in Nigeria due to PLOS Neglected Tropical Diseases The manuscript number is PNTD-D-25-00416,the overall content of the article was good, and the DHIS2 system was able to address the problem of communities using a variety of paper matrices to organize data from the community, which are then transferred to the local government level and then to the district, and finally aggregated to the national level. It was also able to enable many NTD programs to access the timeliness, quality, and availability of comprehensive treatment data during and after MDA campaigns, and can be effective in improving prevention and treatment coverage of neglected tropical diseases.

However, the article also has a bit of a flaw:

1. there are some problems with the formatting, such as the paragraphing on lines 570-572.

--Thank you, we have attempted to fix formatting including tables

2. Another thing is that the references should be in “[]” format instead of “()”, and the format of references should be standardized.

--This has been done

3. At the same time in the article inside the table should be simple and clear, will be some important form of content for the condensation of the table, at the same time will be the table inside the key information for the notes and explanations (such as “*Refresher training” is not explained).

4. references should be adequate so that they strongly support your article.

--We have added additional references.

5. In the study, only 3 states (Jigawa, Enugu, Kwara) are covered, can you state whether this is representative of the diversity of Nigeria as a whole (e.g. difference between North and South). In terms of questionnaire completion: the sample size of the self-administered questionnaire was uneven across states (Kwara=49, Enugu=26), which may affect the credibility of the inter-state comparisons.

--We have provided an explanation for this, as well as highlighted the lack of representation of different states as a limitation of this study

Reviewer #1: 1) Introduction scope – The opening section is clear and instructive, but it presents preventive chemotherapy‑ (PC) NTDs as an uncontested approach. Consider adding a short balanced paragraph (2–3 sentences) outlining key arguments for (programme efficiency, integrated platforms) and against (risk of systematic non‑compliance, limited impact on transmission for some NTDs) large‑scale PC‑MDAs, with citations, and which parts of the MDA does the DHIS2 approach aim to monitor and optimise, and the one it does not (e.g., systematic non-compliance).
I would also add "for" or "against" after "PC" in line 74.

--We have completely reworked the introduction and in line with keeping it concise and relevant to the scope of the research do not see the need for this recommendation

2) Methods organisation – The “Platform and process” to "After the MDA campaign" subsections are dense and largely narrative, perhaps more suited for the methods than for introduction. Also, to save some text, I suggest to convert it into a figure/flowchart (e.g. swimlane‑ diagram) showing data flow: CDD → LGA focal person → State → national DHIS2 instance, including helpline feedback loops.

--We have added a flow diagram showing the platform and process and considerably cyut back on the entire paper.

3) Table 2 ambiguity – The table headings do not make clear whether each row’s number is (e.g., MDA administrators, surveyed households). Later the households become the denominator for coverage verification, but “NA” appears in the same column. If applicable, i suggest splitting the information: (i) sample characteristics of MDA staff (if relevant) and (ii) household survey frame. Clearly state who each number represents and ensure consistency. Moreover, Table 2 seems best suited for the start of the results section, particularly in the first sentence of the results.

--Thank you, we have updated the tables

4) The rationale for choosing the three States within Nigeria and the two different partner‑support models is mentioned, but the criteria are not spelled out. Please add, if reasoning available, a brief paragraph explaining why these particular settings were considered representative (and what variation they capture).

--Thank you, we have added more information on this

5) The quantitative analysis is described in three paragraphs (lines 296‑313) with no mention of significance threshold (95%CI?), clustering adjustments (if any). To note that the coverage surveys interview individuals nested within households, which are themselves nested within communities/LGAs. If you analyse the raw person‑level data as though every observation were independent, the standard errors (and thus confidence intervals and p‑values) will be too small, because responses from the same household or community are correlated. Clustered standard errors may correct for this by telling the software that observations share a common error structure within each cluster (e.g. household ID/community-ID). In Stata this is as simple as adding , vce(cluster hhid) to a regression or proportion command.

--Given the scope and main objective of this manuscript we don’t feel we should add a great deal of additional detail on the coverage evaluation surveys (CES), however we have included a description of methods used based on already standard WHO CES for MDA programmes.

6) Because DHIS2 instances can contain personally identifiable information, consider clarifying whether any personal data (names, phone numbers) were stored on the study server and, if so, how they were protected.

--The DHIS2 data recorded did not contain personally identifiable information - treatment data at the community level was the lowest level input in the system.

Reviewer #2: The manuscript lacks a sentence specifically stated the primary and secondary objectives. Stating a measurable primary hypothesis would strenghthen the focus. A mixed-methods, pre-post comparison is suitable. However, most of the data are qualitative and subjective indicator are used. Objective pre- vs. post-metrics (such as coverage, stock accuracy, timeliness) that the introduction highlights are large absent or anecdotal. The DHIS2 system was piloted and rolled out in three states. The rationale why the study states were selected remains unclear. The sample counts are reported for surveys, yet no power or saturation justifications is provided. Descriptive statistics are shown; however, no inferential tests are applied to pre-post comparisons, which may weaken the conclusions about improvements by leaving them without statistical support. In terms of ethical concerns, the authors state that Nigeria's National Health Research Ethics Committee granted a waiver. No overt ethical issues are evident.

**Results:**

Reviewer #1: 7) In Figure 1, Put xaxis label (“State”) and ‑yaxis‑ label (“Number of trained users who accessed DHIS2”) directly on the graphic. Mention the figure at its first logical point (in line ~336: “93 % in Enugu…”).

--Thank you, we have updated the tables

8) Coverage inconsistency analysis – The discussion notes mismatches between DHIS2 and coverage survey but stops short of analyzing why. Could this discrepancies stem from numerator inflation/deflation, denominator mis‑estimation, or delayed data synchronization. This could make recommendations more actionable, as it is one of the major challenges of MDA's monitoring programmes.

--This has been addressed in the discussion section

9) Table 3 text - Consider briefly stating the main categories of issues if relevant (stockouts, duplicate records, etc.) and give one illustrative example of resolution per category. A brief paragraph interpreting the pattern (e.g. why drug inventory issues dominated in Kwara) would help the reader; otherwise the table remains descriptive.

--Again we have refocused this manuscript away from drug stocking and instead kept it aligned with the revised objectives.

10) Large negative value in Table 4 – The EnuguIsi Uzo difference (−21.85 pp) appears very large‑. Re‑check the calculation, and in all tables fix numbers to one decimal place for consistency (or other place, as authors prefer). Consider adding numerator/denominator of coverages in a supplementary appendix, as this can be relevant to understand the importance of difference in coverage between DHIS2 and coverage surveys.

--This has been updated, thank you

11) Table 5 formatting Explain why some rows are bold (e.g. p < 0.05) in a footnote.

--This is due to statistical significance, and have been updated in the table

Reviewer #2: The analysis partially matches the analysis plan. The manuscripts outlines a mixed-methods evaluation. The analysis of MDA treatment and stocking reporting is largelly based on users' perception, which is somewhat subjective. Few quantitative estimates are applied to demonstrate the effect of DHIS2 platform on improvement. Objective indicators such as drug stock trakcing, inaccurate coverage reportings. This may limit the strength of the conclusion.
The results are moderately clearly and completely presented. The qualitative results are clearly structured by theme and supported with quotations. Yet, more analytical techniques, such as code-code cooccurrence and thematic analysis should be employed to deepen the interpretation. If possible, the authors should use objective estimates for key outcomes--such as treatment coverage, drug usage, and stock wastage, etc--to strengthen the validity fo their findings and reduce reliance on self-reported or perceived improvements.
The quality and clarity of figures and tables need improvement. Although the tables and figures are informative, there are occasional inconsistencies or lack of clarity. Some figures would benefit from reformatting.

**Conclusions:**

Reviewer #1: The paper provides one of the first empirical assessments of DHIS2 for real-time‑ MDA stock and treatment reporting in an African setting. With the clarifications above, it will serve as a useful operational blueprint for other NTD programmes and digital health‑ implementers.

12) Definition of “treated” – Although informative, a minor limitation of the study regarding coverage would be the definition of "swallowed" as this was self-reported and may be subject to desirability bias. Consider also other potential biases and limitations, such as Selection bias – States were purposively chosen; results may not extrapolate nationally; Short followup‑ – The study captures one MDA round per State; sustainability over multiple cycles is untested; Incomplete cost analysis – No costing of data bundles, helpline staff time or device purchase; important for scale‑up.

--We have cut back on this section and created a study limitation section to address these concerns

13) The manuscript notes that Jigawa outperformed other States on ownership scores, but does not explore whether partner intensity (pilot study), prior DHIS2 exposure or baseline capacity contributed to this difference.

--We have re-calculated the ownership scores, which initially compared user expectation versus post-user experience, to now compare their perception of prior MDA versus the recent MDA where the platform was deployed. Consequently, all the states report similar patterns of improvement.

Reviewer #2: The conclusions are partially supported by the data presented. The manuscript effectively summarizes user-reported improvements in data access, usage, and perceived ownership. Yet many of these conclusions are based on qualitative feedback without corresponding objective measures. The limitations of the analysis--particularly the reliance on subjective data and lack of inferential test/interpretive analysi-- should be more explicitly discussed.
The public health relevance is clearly addressed, as the study is positioned within efforts to improve programmatic efficiency and disease control through better data system. Clarifying the connection between improved data processes and their impact on health outcomes would further reinforce the public health significance of the study.

--xxx

**Editorial and Data Presentation Modifications?**

Reviewer #1: 14) Possible redundancy – 9 main tables + 1 figure and abundant descriptions crowd the methodology and results section; some may be best suited to supplementary material. Consider moving some tables to supplementary appendix; while Retaining main results in the text and refer readers to the appendix.

--Thank you, we have updated the tables

15) Table 1 - Add an abbreviations footnote (e.g. NW, SE, SW, MDA, LGA, CBM, etc.).

--Done

16) Section numbering - Introduce hierarchical numbering (e.g. 3 Results, 3.1 DHIS2 meta‑data, 3.2 Helpline monitoring, 3.3 Treatment coverage data, 3.4 field diaries, 3.4.1 accessibility of data) to help navigation.

--We have updated the sections with numberings

17) The manuscript is generally well written. However, as mentioned before, consider trimming lengthy methodological/results detail and moving it to Supplementary Methods. The manuscript currently reads half scientific article, half programme report.

--We have trimmed the length and shifted the focus of the manuscript.

18) correct "meticizan" in line 489 to "mectizan"; "log shows" to "log showed" in line 351;

--Done

19) a couple of more references in the discussion could be beneficial.

--We have added additional references.

Reviewer #2: Several tables and figures require reformatting to improve clarity and ensure consistency throughout the manuscript.

--Thank you, we have updated the tables and figures

**Summary and General Comments:**

Reviewer #1: The study by Imhansoloeva et al. tackles a high‑priority operational challenge for national neglected‑tropical‑disease (NTD) programmes: obtaining timely, reliable treatment‑coverage and drug‑stock data during mass‑drug administration (MDA). The mixed‑methods design (DHIS2 metadata analytics, helpline monitoring, treatment‑coverage verification and field diaries) is appropriate and, in places, impressively granular. Nevertheless, some clarifications and streamlining steps are needed before the manuscript is ready for publication.

--Thank you and we hope the revisions help with these concerns.

Reviewer #2: This manuscript addresses a timely and relevant topic: the use of the DHIS2 platform to support mass drug administration (MDA) for neglected tropical diseases (NTDs) in high-disease burden settings. The research is grounded in a solid background, with a clear public health rationale and alignment with global NTD elimination goals. The integration of both qualitative and descriptive quantitative data provide useful insights into user experience, system adoption and perceived improvements in data accessibility, quality and owenership, etc.
However, the presentation of the data requires improvement. Greater use of structured formatting, clearer legends, and visual enhancements would enhance clarity and impact. In addition, there is also a gap between the study's stated objectives and the data actually analyzed. These outcomes are primarily discussed through qualitative user perceptions, with limited use of objective estimates. While the qualitative findings are well-organized and supported by quotations, the analysis would benefit from deeper qualitative analytical teniques. There is no concern regarding research ethics.

--Thank you and we hope the revisions help with these concerns.
